# Corrosion Behavior of Coated Low Carbon Steel in a Simulated PEMFC Environment

**DOI:** 10.3390/ma16083056

**Published:** 2023-04-12

**Authors:** Diana Nicoleta Avram, Corneliu Mircea Davidescu, Iosif Hulka, Mircea Laurentiu Dan, Elena Manuela Stanciu, Alexandru Pascu, Julia Claudia Mirza-Rosca

**Affiliations:** 1 Faculty of Industrial Chemistry and Environmental Engineering, Politehnica University Timisoara, 2 Piata Victoriei, 300006 Timisoara, Romania; 2 Renewable Energy Research Institute—ICER, Politehnica University Timisoara, 138 Gavril Musicescu Street, 300774 Timisoara, Romania; 3 Materials Engineering and Welding Department, Transilvania University of Brasov, 29 Eroilor Blvd., 500036 Brasov, Romania; alexandru.pascu@unitbv.ro; 4 Department of Mechanical Engineering, Las Palmas de Gran Canaria University, 35017 Las Palmas de Gran Canaria, Spain

**Keywords:** electrochemical evaluation, protective coatings, PEMFC, corrosion, laser cladding

## Abstract

Here, potential metallic bipolar plate (BP) materials were manufactured by laser coating NiCr-based alloys with different Ti additions on low carbon steel substrates. The titanium content within the coating varied between 1.5 and 12.5 wt%. Our present study focussed on electrochemically testing the laser cladded samples in a milder solution. The electrolyte used for all of the electrochemical tests consisted of a 0.1 M Na_2_SO_4_ solution (acidulated with H_2_SO_4_ at pH = 5) with the addition of 0.1 ppm F^−^. The corrosion resistance properties of the laser-cladded samples was evaluated using an electrochemical protocol, which consisted of the open circuit potential (OCP), electrochemical impedance spectroscopy (EIS) measurements, and potentiodynamic polarization, followed by potentiostatic polarization under simulated proton exchange membrane fuel cell (PEMFC) anodic and cathodic environments for 6 h each. After the samples were subjected to potentiostatic polarization, the EIS measurements and potentiodynamic polarization were repeated. The microstructure and chemical composition of the laser cladded samples were investigated by scanning electron microscopy (SEM) combined with energy-dispersive X-ray spectroscopy (EDX) analysis.

## 1. Introduction

Without the natural greenhouse effect (GHG) regulating the planet’s atmospheric temperature, life as it exists today would not be possible. The atmosphere retains enough solar energy to maintain an average global temperature of 14 °C due to the natural existence of GHGs on Earth. Carbon dioxide (CO_2_), methane (CH_4_), ozone (O_3_), nitrous oxide (N_2_O), and steam (H_2_O) are the main greenhouse gases that are naturally recirculated in the atmosphere. Since the Industrial Revolution, increases in the concentrations of the main GHGs have been recorded [1]. Human interference has brought considerable changes to the natural landscape, because of activities such as deforestation and burning fossil fuels for transportation and heating, agricultural, and industrial activities. This has augmented the natural greenhouse effect, with CO_2_ being added in voluminous amounts in the Earth’s atmosphere [2,3,4]. The U.S. Energy Information Administration (EIA) reported that, from 1980 to 2012, CO_2_ emissions and primary energy consumption grew at an average annual increase of 1.7% and 2%, respectively. In this period, CO_2_ emissions grew by 75% and the global primary consumption of energy increased by85% [5]. Nowadays, replacing conventional energy sources with cleaner energy alternatives has become a priority. Anthropogenic GHG emissions can be reduced with the use of renewable energy sources, such as wind, solar, hydropower, geothermal, biomass, and fuel cells [6,7].

Among these green technologies, the fuel cell is regarded as a promising solution to the energy crisis and a key to combat the global warming phenomena. The advantages of using fuel cells include a high power density, silent operation, good performance, and low or even zero emissions if the fuel is from a renewable source. Among the different types of fuel cells, PEMFCs have attracted a lot of interest in the scientific community because the technology can be implemented in different sectors, from portable electronics to power generation systems [7,8,9]. Over the past years, intense research has been done on implementing the PEMFCs systems in the automotive sector. Several prototypes of fuel cell vehicles (FCV) have been successfully manufactured, such as Toyota Mirai, Hyundai ix35, Daimler F-Cell, and Honda FCX-Clarity. The great potential of fuel cell applications has also extended to various vehicles such as buses, trucks, forklifts, and airport transportation vehicles. However, the commercialization of such systems has two main obstacles, which are their low durability and high price. Although substantial advancements have been made in recent years, these previously mentioned hurdles still impose a challenge in the current technologies [7,10].

One of the key components that could determine a better performance for PEMFCs is represented by the bipolar plate. BP interconnects the cathode of a cell with the anode of the adjacent cell, forming a stack. To achieve adequate power output, several individual cells are connected in series to form a fuel cell stack. This means that BPs represent a considerable fraction of the weight and cost of the stack. Other functions of the BPs include (i) homogenous distribution of reactant gases within the flow channels to the active sites, (ii) management of water, and (iii) heat transfer, ensuring the electrical flow within the cell and offering mechanical stability to the stack. To fulfill all of the requirements, BP materials must combine a suitable mechanical strength with a high corrosion resistance, together with a good electrical and thermal conductivity [11,12]. High density graphite, polymer-based composites and metals are the main materials used for the development of BPs. Among the three, metallic BPs have attracted a lot of interest in the research community due to their superior mechanical and physical properties. Various metals and metallic alloys, such as stainless steel [13,14], aluminum [15], mild steel [16,17,18], copper [12], titanium [13,19], aluminum alloys [20], copper alloys [21], titanium alloys [22], and nickel alloys [23,24], have been studied as possible candidates for BPs materials. Mild steels have the advantage of being inexpensive materials with excellent mechanical properties, but with a weak corrosion resistance in the acidic PEMFCs environment. Different surface modification techniques or applying protective coatings can increase the corrosion resistance of low-carbon steels [12]. In their research, Bai et al. [16,17] reported that chromized-AISI 1045 could be a good alternative BP material for PEMFC applications. Various test analyses have been used to evaluate the performance of chromized-AISI 1045, and the results show that it is comparable to that of stainless steel or graphite. Previous work [25] has included studies on surface modification AISI-1020 with a chromized coating containing carbides and nitrides, with promising results for PEMFC applications. Yuan et al. [18] reported that the corrosion resistance of nickel coated AISI 1045 was improved after low temperature pack chromizing and that it is a good alternative material for PEMFC applications. 

In our previous studies [26,27], we successfully laser cladded NiCr(Ti)-based coatings onto low carbon steel plates and electrochemically tested them in a very acidic PEMFC environment (aggressive conditions). The NiCr-based superalloy was selected as the base material due to its good corrosion resistance [28], while titanium was added as a reinforcing material into the composition (up to 10 wt% Ti addition). From the literature and applications, strong acidic solutions used as an electrolyte have a great influence on the corrosion of metallic BPs. Lædre et al. [29] showed that very low pH values can change the composition and thickness of the oxide layer in such a way that it may not exist in an operating PEMFC. This is also supported by the research of Feng et al. [30], who concluded that using mild conditions, instead of aggressive conditions (solutions such as 1 or 0.5 M H_2_SO_4_), for electrochemical testing could simulate the PEMFC working environment better, while the research of Hinds et al. [31] showed that in situ measurements of pH on the exhaust water from fuel cell stacks was in the range of 3–4 for the cathode and 5–7 for the anode. 

In this regard, our present study focused on electrochemically testing the laser cladded materials in a milder solution. The electrolyte used for all of the experiments was Na_2_SO_4_ 0.1 M (acidulated with H_2_SO_4_ at pH = 5) + 0.1 ppm F^-^. Furthermore, the titanium content within the coating from 1.5 to 12.5 wt% Ti was added, and the variation in titanium was studied in terms of the microstructure and corrosion resistance. The morphology and chemical composition of the laser cladded samples were investigated by SEM combined with EDX analysis. The electrochemical protocol consisted of OCP and EIS measurements, potentiodynamic polarization, followed by potentiostatic polarization under simulated PEMFC anodic and cathodic environments for 6 h each. After the samples were subjected to potentiostatic polarization, the EIS measurements and potentiodynamic polarization were repeated.

## 2. Materials and Methods

### 2.1. Materials and Deposition Process

Feedstock powders of MetcoClad 625F (NiCr-based powder) and Metco 4010A (Ti powder, 99% purity) from Oerlikon Metco Switzerland were used as the laser-cladding materials. AISI 1010 plates with dimensions of 60 × 25 × 5 mm were used as the substrates for laser cladding NiCr-based alloys (designated B in our manuscript) with different Ti additions. The titanium content within the coating varied as follows: 1.5, 3, 5, 7, 10, and 12.5 wt% Ti. The chemical compositions of the feedstock powders and for the low carbon steel were the same as those presented by the manufacturers [32,33,34]. 

**Figure 1:**
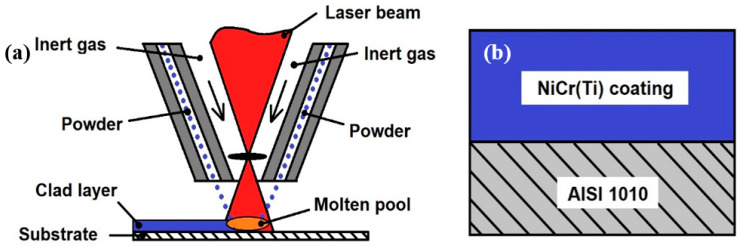
Schematic representation of (**a**) the laser-cladded process and (**b**) protective coatings.

The equipment used for powder deposition and the deposition parameters were as presented in our previous study [26]. In summary, a diode laser with λ = 975 nm (Coherent F1000) mounted on a robotic arm (Cloos 6-axes) was used for laser cladding of feedstock powders fed from a processing head (Precitec WC 50) onto the AISI 1010 substrate. The carrier gas (argon) was used in a feeding system (Termach AT-1200 HPHV) to transport the powders to the processing head. The process parameters used for laser cladding deposition included preheating the feedstock powder, and using an 859 W laser power with a 40 cm/min deposition speed and 15.1 L/min argon flow. Ten partially overlapped tracks were deposited onto the AISI 1010 substrate with an 45% overlap degree between the subsequent tracks. The schematic representation is shown in Figure 1.

### 2.2. Characterization of Protective Coatings

The microstructure of the laser-cladded coatings was investigated in cross section at a low magnification (1000×) using a scanning electron microscope (FESEM Zeiss Sigma 300 VP, Carl Zeiss, Jena, Germany). To reveal the microstructure of the coatings produced by laser cladding at a high magnification (30,000×), the samples were examined by FESEM (Quanta FEG 250, FEI, Hillsboro, OR, USA) using backscattered electron detector (BSD). Elemental analysis was performed as well using energy dispersive X-ray spectroscopy (EDX) with an Apollo SSD detector (EDAX Inc. Mahwah, NJ, USA).

A Future-Tech FM-700 microhardness tester with a Vickers indenter (Future-tech Corp, Kanagawa, Japan) was used for the microhardness measurements. Ten HV_01_ indentations were performed on each coating in the cross-section starting from the top towards the substrate; two measurements were performed in the heat affected zone (HAZ) and another two were performed in the substrate. For the measurements, a load of 100 gf and a dwell time of 15 s were employed for each indentation. 

A three-electrode system was used for the electrochemical evaluation of the laser cladded samples in a solution consisting of 0.1 M Na_2_SO_4_ (acidulated with H_2_SO_4_ at pH = 5) with the addition of 0.1 ppm F^-^. The corrosion behavior of the samples was tested using a potentiostat/galvanostat SP150 (BioLogic Science Instruments, Seyssinet-Pariset, France). The samples were placed in a corrosion cell with an exposed area of 1 cm^2^, together with a platinum mesh and an Ag/AgCl electrode acting as the counter and reference electrode, respectively. Different grades of SiC abrasive papers (grit size from 120 to 2400) and a 3 μm diamond suspension were used to grind and polish the samples until they had a mirror-like surface. A solution of 10% oxalic acid was used to electrolytically etch the samples for 12 s at 3 V to visualize the microstructure of the laser-cladded coatings. Afterward, distilled water and ethanol were used to clean the samples. 

The electrochemical evaluation protocol consisted of OCP and EIS measurements, followed by potentiodynamic and potentiostatic polarization. After the samples were subjected to potentiostatic polarization, the EIS measurements and potentiodynamic polarization were repeated. EIS measurements were employed with the impedance module of SP150, as described in our previous paper [26], in the frequency range of 10^−1^–10^5^ Hz with a 10 mV AC voltage amplitude. A ZView 3.4 software (Scribner Associates, Inc., Southern Pines, NC, USA) was used to fit all of the experimental EIS data to their corresponding equivalent electrical circuit (EEC). Potentiodynamic polarization curves were recorded in the potential range of ±0.250 V at a scan rate of 1 mV·s^−1^. Potentiostatic polarization curves were employed to simulate the cathodic and the anodic PEMFC environments. In this measurement, potentials of +0.736 V and −0.493 V were applied to the electrochemical configuration, respectively, and they were maintained for 6 h each. Afterwards, the EIS measurements and potentiodynamic polarization were repeated in the same procedure, as previously described.

## 3. Results and Discussions

### 3.1. Microstructure and EDX Analysis of Cladded Coatings

**Figure 2:**
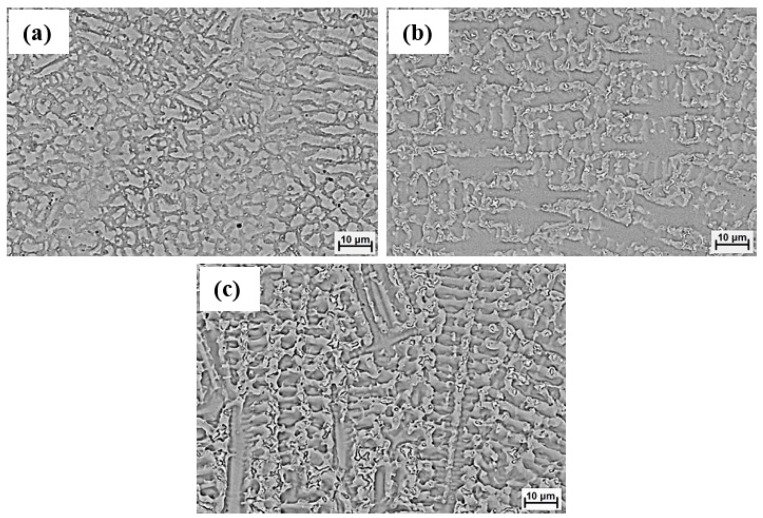
SEM images of laser-cladded coatings with different Ti contents after etching with oxalic acid: (**a**) B, (**b**) B + 5% Ti, and (**c**) B + 10% Ti.

Representative SEM images of the laser-cladded samples were taken from the cross-section for coatings without Ti and with the addition of 5 and 10 wt% Ti, and are presented in Figure 2a–c. It can be noticed that there were no significant defects, such as cracks or pores, across the coatings. The etchant revealed a dendritic structure for all of the laser cladded coatings. Furthermore, it can be seen that as the Ti content increasesd within the coating, the size of the dendrites increased. 

**Figure 3:**
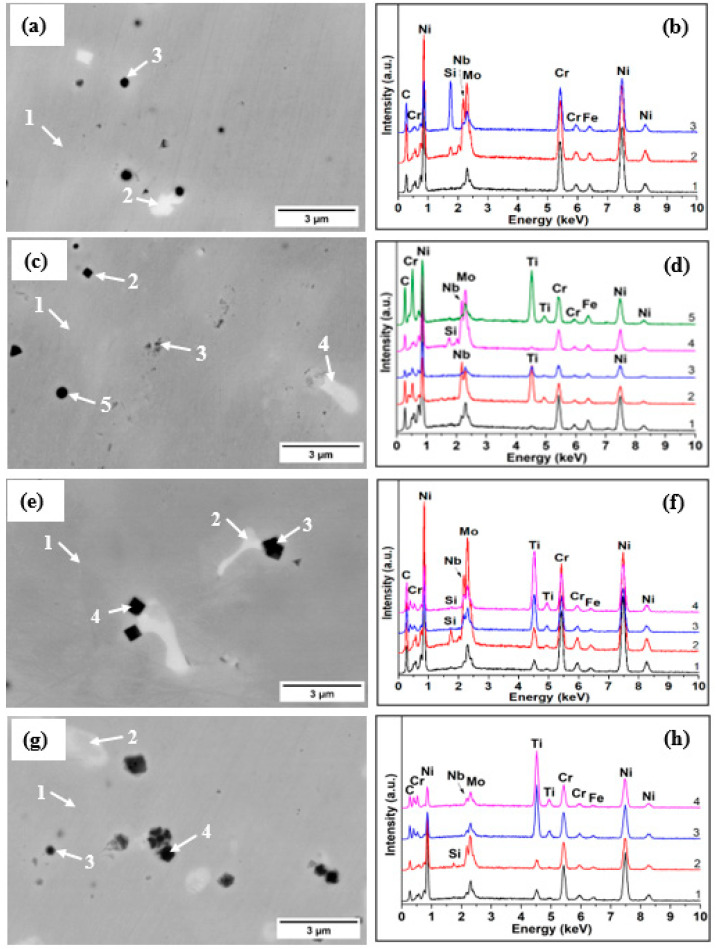
SEM images with the corresponding EDX spectra of coatings with different Ti contents: (**a**,**b**) B, (**c**,**d**) B + 1.5% Ti, (**e**,**f**) B + 5% Ti, and (**g**,**h**) B + 12% Ti.

In our previous study, it was observed that the coatings consisted mainly of γ-Ni-Cr phase with Ti present at 51.59° 2θ, which dissolved into the FCC Ni-Cr phase. Moreover, no other peaks associated with other precipitates of Ni-Cr alloys such M6C, MC and M23C6 carbides were identified due to their low intensity [26]. Generally, these precipitates developed at grain boundaries and inter-dendritic areas during solidification and within the coating, increasing the cracking susceptibility at the fusion zone, reducing its corrosion resistance and ductility [35]. To investigate the precipitates, EDX spot analysis was performed on an un-etched laser-cladded coating at high magnification representative micrographs, as presented in Figure 3. It is well known that SEM analysis using BSD is used to detect elastically scattered electrons, delivering micrographs with information about the composition. Thus, the number of back scattered electrons reaching the detector is strongly related to the atomic number of the elements. The higher the atomic number, the brighter the element appears in the image.

Generally, three main areas could be observed in the cross-sectional micrographs of the investigated coatings (i) a gray area, which represents the Ni-Cr matrix; (ii) a lighter phase rich in Nb and Mo, which represent laves; and (iii) dark areas with a high Si content (for the coating without Ti) and high Ti content (for the coatings manufactured with Ti addition), which represent MC carbides. During deposition, Ti powder particles dissolve in the molten pool and react with C atoms, leading to the formation of TiC when solidification takes place [36]. Ti particles precipitated during solidification generally in spherical and rhombus shapes distributed randomly within the coatings. Lei et al. also reported that a rhombus morphology for TiC precipitates wasformed during the deposition of TiC/NiCrBSiC composite carbides using laser cladding [37]. From the images, it can be observed that the size of carbides increased with the addition of Ti within the coatings. 

### 3.2. Microhardness of the Coatings

**Figure 4:**
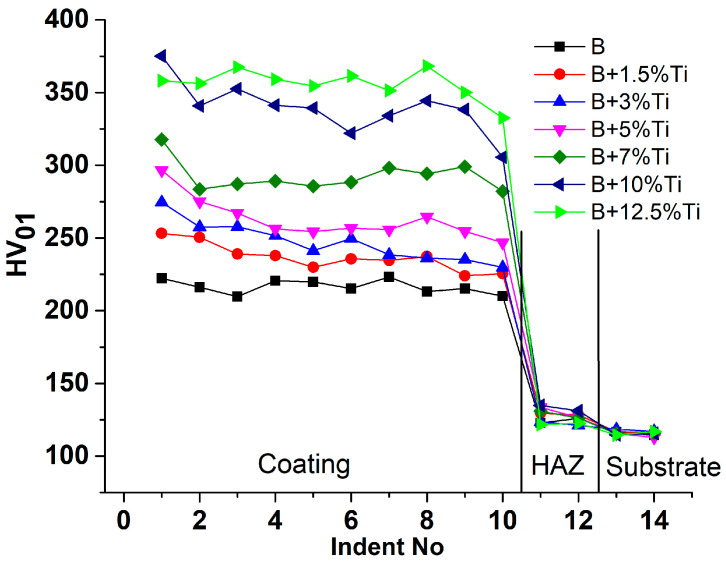
Microhardness values measured in the cross-section from the top of the coatings to the substrate.

Figure 4 represents the microhardness comparison of the investigated coatings. The microhardness values were measured across the cross-section of the coatings, starting from the top of the coatings down to the substrate. It can be seen from the graph that the coatings manufactured under the same process parameters have great differences in their function after the addition of Ti. The increase in hardness values were attributed to the formation of hard phases within the coatings. With the increase in Ti content, the number of hard phases increased, which directly led to an increase in hardness. Consequently, the coating without the addition of Ti presented the lowest hardness, while the coating with the addition of 12.5% Ti presented the highest hardness values. The fluctuation in the measurements was caused by the randomly distributed hard phases within the coatings. HAZ had a somewhat higher hardness than the substrate, caused by the metallurgical changes that occurred as a result of the fast heating−cooling process that took place during cladding.

### 3.3. Electrochemical Evaluation before the Durability Test

**Figure 5:**
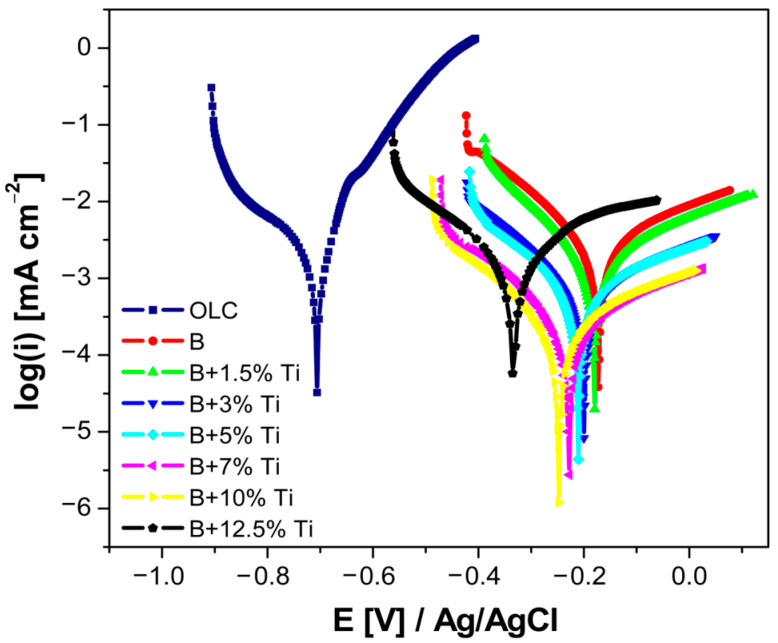
Potentiodynamic polarization curves recorded on all laser-cladded samples, before potentiostatic polarization, at a scan rate of 1 mV·s^−1^.

Corrosion resistance in a simulated PEMFC environment is an important criterion when evaluating the performance of a potential BPs material. Potentiodynamic polarization curves of the substrate and laser cladded coatings with various Ti contents were recorded at a scan rate of 1 mV·s^−1^, before submitting the samples to a potentiostatic polarization test. Figure 5 presents the Tafel plots of the substrate and laser-cladded samples tested in Na_2_SO_4_ 0.1 M + 0.1 ppm F^-^ at room temperature, before measuring the current transients in anodic and cathodic environments. From the potentiodynamic curves, it can be observed that all of the coatings presented an enhanced corrosion resistance compared with the substrate. Comparing the coatings, a slight negative shift in the corrosion potential with the increase in Ti content was observed.

Using the Tafel extrapolation method, the corrosion current density (i_corr_) and corrosion potential (E_corr_), the anodic and cathodic Tafel slopes were calculated and are presented in Table 1. From the polarization parameters, it can be seen that the current density had the highest value for the substrate. Moreover, the results indicate that the current density values recorded for the coatings decreased with increase in titanium addition, up to the addition of 10 wt% Ti. On the other hand, for the laser-cladded coating with the addition of 12.5 wt% Ti, a sudden increase in the current density values was found.

**Figure 6:**
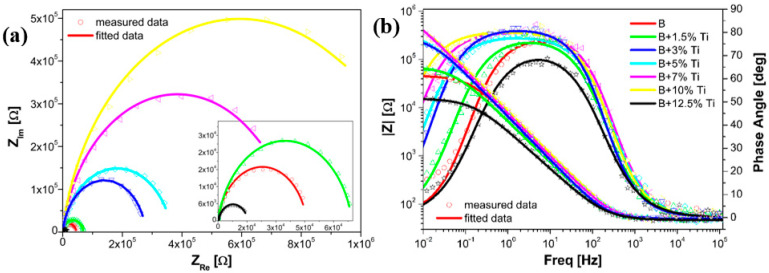
Nyquist (**a**) and Bode (**b**) diagrams recorded on all laser cladded samples before potentiostatic polarization.

Based on the polarization curves and Tafel parameters, the coatings showed good corrosion results up until the addition of 10 wt% Ti. The results were supported by EIS measurements conducted using the open circuit potential value (Figure 6). In the Nyquist plots (Figure 6a), all laser-cladded samples showed a single capacitive loop, with a substantial increment in the semicircle radius as the Ti content increased within the coating, up until the addition of 10 wt% Ti. Then, a sudden decrease was noticed. A low spectra diameter was obtained for the coating with the addition of 12.5 wt% Ti. 

Bode plots of the laser-cladded coatings are presented in Figure 6b. As the Ti addition increased within the coating, the Bode-|Z| diagrams showed higher impedance modulus values, while the Bode-phase diagrams showed higher phase angle plots. These results could be attributed to the development of a passive film on the surface of the coating with a growth of the corrosion resistance. A sudden decrease in the impedance modulus values and phase angle plots were noticed for laser-cladded coating with the addition of 12.5% Ti. As the sample with 12.5 wt% Ti content showed a lower corrosion resistance than the rest of the coatings, the current transients were measured only for laser-cladded samples up until the addition of 10 wt% Ti. The repeated EIS and Tafel plots were used to characterize the coatings after the durability test.

**Figure 7:**
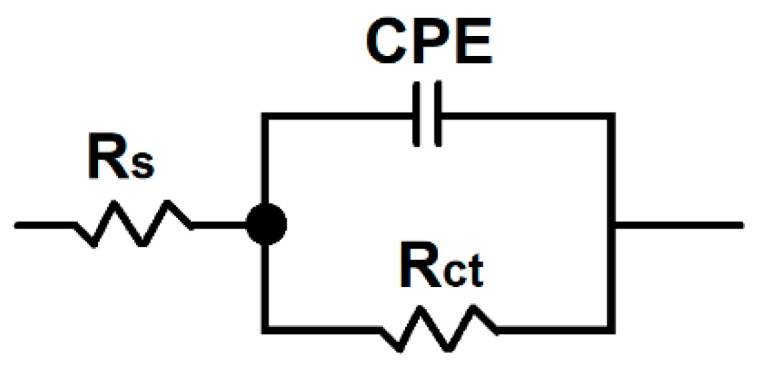
Electrical equivalent circuit used for the spectra fitting.

The EIS data were fitted using the equivalent electric circuit shown in Figure 7, and the calculated values of the circuit elements used for modeling the samples are presented in Table 2. In EEC, R_s_ stands for the solution resistance between the working and reference electrodes, R_ct_ represents the charge transfer resistance at the coating/electrolyte interface, and CPE indicates the constant phase element. From the results shown in Table 2, similar values of solution resistance were obtained for all of the samples, indicating the same ion conductivity in the test solution. As the Ti content increased within the coating, the charge transfer resistance had higher values, indicating a lower reaction rate.

**Figure 8:**
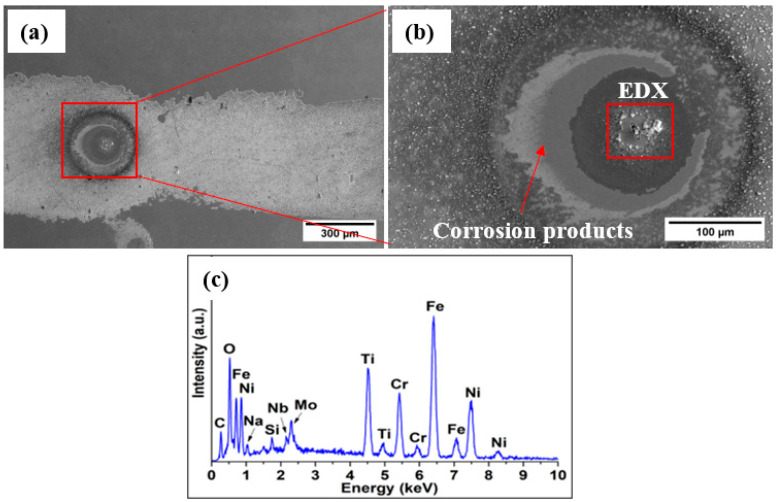
Surface morphology of the coating manufactured with 12.5% Ti exposed to the electrolyte: (**a**) corrosion products, (**b**) corrosion products and micro-crack, and (**c**) EDX spectra reveling the presence of Fe and O on the surface of the coating.

To investigate the lower corrosion resistance of the coating manufactured with the addition of 12.5% Ti after exposure to the electrolyte, the surface was analyzed using SEM. According to the micrographs presented in Figure 8, the surface contained corrosion products and micro-cracks. The EDX spectra collected in the area marked with the red rectangle in Figure 8b show the presence of Fe and O. The increased amount of Ti led to the formation of micro-cracks within the coatings, which allowed the electrolyte to reach the substrate [38]. The micro-cracks appeared due to the residual stress caused by the thermal and chemical effects [39], attributed to the rapid cooling and to the higher amount of Ti, leading to a lower corrosion resistance.

### 3.4. Potentiostatic Polarization

**Figure 9:**
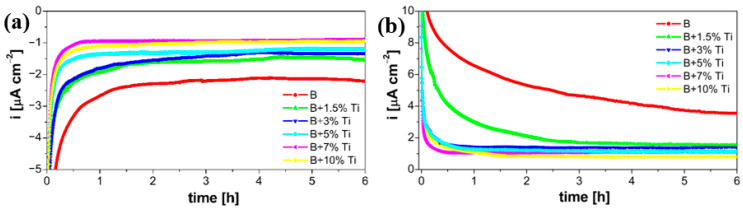
Potentiostatic polarization curves of laser-cladded coatings recorded for 6 h, at a constant potential of: (**a**) +0.736 V and (**b**) −0.493 V.

The durability of laser-cladded coatings in simulated cathodic and anodic PEMFC environments were considered by measuring the current transients of the samples at a constant potential, as a function of time. Figure 9 shows the potentiostatic polarization curves of every coating, measured for 6 h, at a constant potential of +0.736 V and −0.493 V, which corresponded to the release of oxygen and hydrogen, respectively. Figure 9a presents the current transients measured in the cathodic PEMFC environment. It can be seen that the current density of all of the coated samples initially increased from a negative direction and then gradually stabilized at low negative values. The negative current density values could be explained because the corrosion potential of the laser-coated samples was more positive than the applied potential. Based on this, the negative values were attributed to a reduction in H^+^ ions, which changed to H_2_, thus providing cathodic protection to the laser-coated samples. During potentiostatic polarization, all of the samples displayed a stable corrosion current density. The cathodic current density obtained for the B coating was around −2.21 µA·cm^−2^, while the laser-cladded coatings with Ti additions showed lower values of between −1.55 and −0.89 µA·cm^−2^. Figure 9b presents the current transients measured in the anodic PEMFC environment. It can be seen that the current density of all of the coated samples initially decreased from a positive direction and then gradually stabilized at low positive values. This behavior could be explained by the formation of a passive film on the surface of the samples. It is clear that the current densities decreased with the increase in Ti content within the coating. During the measurement, all of the samples displayed a stable corrosion current density. The anodic current density obtained for the B coating was around 3.55 µA·cm^−2^, while the laser-cladded coatings with Ti additions showed lower values of between 1.52 and 0.81 µA·cm^−2^. From the presented results, the NiCr-based coating with the addition of 10 wt% Ti showed the lowest current density in both the cathodic and anodic environment.

### 3.5. Electrochemical Evaluation after Durability Test

**Figure 10:**
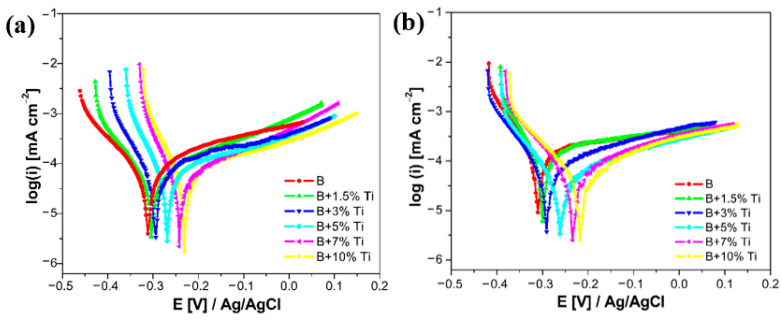
Potentiodynamic polarization curves recorded at a 1 mV·s^−1^ scan rate on laser-cladded samples: (**a**) after cathodic and (**b**) anodic potentiostatic polarization.

Figure 10 presents the potentiodynamic polarization curves of the laser-cladded samples with additions of up to 10 wt% Ti content, after measuring the current transients in the simulated PEMFC environment. Using the Tafel extrapolation method, the polarization parameters of the laser-cladded samples are calculated and listed in Table 3. From the polarization parameters, the corrosion current densities (i_corr_) of the laser-cladded coatings, in both the cathodic and anodic environments, had lower values with the increase in titanium content. On the other hand, the polarization resistance (R_p_) in both cases had higher values for coatings with a greater Ti content. After simulating the PEMFC cathodic environment (Figure 10a), the corrosion potential of the laser-cladded samples had a slight positive shift with the increase in Ti content. All of the laser-cladded coatings with the addition of Ti had a higher corrosion potential (E_corr_) that the B coating. The same behavior of the corrosion potential could be observed after simulating the PEMFC anodic environment (Figure 10b).

Comparing the Tafel plots before and after potentiostatic polarization, a negative shift on the corrosion potential for samples up to 5% Ti content, in both cathodic and anodic environments, can be seen. On the other hand, in both environments, a small positive shift in the corrosion potential for coatings with the addition of 7 wt% and 10 wt% Ti was observed. Comparing the polarization parameters before and after potentiostatic polarization, an increase in the corrosion current density for all of the laser-cladded samples was noticed, but the values were all lower than the DOE’s target for 2020, which is 1 µA·cm^−2^.

**Figure 11:**
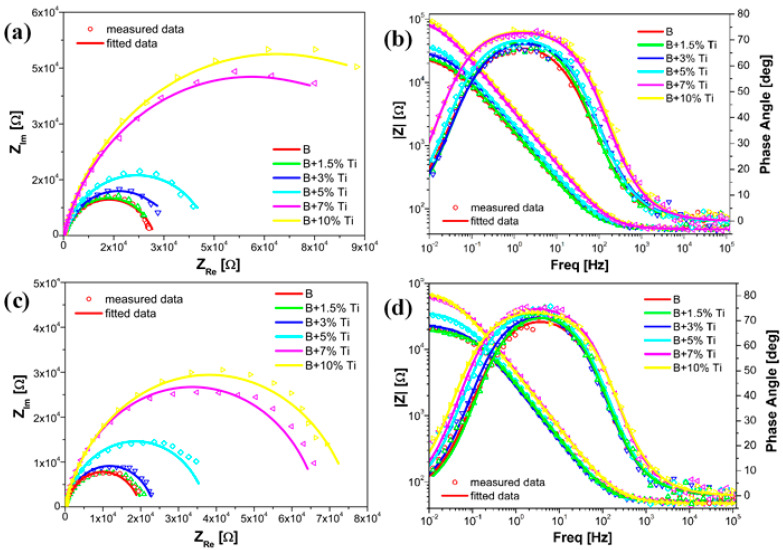
EIS measurements recorded on laser-cladded samples: (**a**,**b**) after cathodic and (**c**,**d**) anodic potentiostatic polarization.

Figure 11 shows the Nyquist and Bode plots for the coated samples after measuring the current transients. From the Nyquist plots, in both the cathodic and anodic environments (Figure 11a,c), all of the laser-cladded samples showed a single capacitive loop, with a substantial increment in the semicircle radius as the Ti content increased within the coating. In both cases, a low spectra diameter was obtained for coatings without the addition of Ti and the highest spectra diameter was recorded for coatings with the addition of 10 wt% Ti. The Bode plots in both the cathodic and anodic environments are presented in Figure 11b,d, respectively. In both cases, the Bode-|Z| diagrams showed higher impedance modulus values and higher Bode-phase angle plots as the Ti content increased within the coating.

Comparing the Nyquist plots before and after potentiostatic polarization, in both environments, the diameter of the semicircle decreased substantially after the durability test. Furthermore, after potentiostatic polarization, the Bode-|Z| and Bode-phase angle diagrams showed lower impedance modulus values and phase angle plots, respectively. The EIS data, after potentiostatic polarization, were fitted using the same equivalent circuit model, as presented in Figure 7. From the calculated values of the circuit elements after potentiostatic polarization, as shown in Table 4, similar values for the solution resistance were obtained for all of the samples, in both environments. Furthermore, as the Ti content increased within the coating, the charge transfer resistance had higher values. On the other hand, when comparing the results from the EEC before and after potentiostatic polarization, in both environments, a decrease in the charge transfer resistance values was noticed.

## 4. Conclusions

Potential metallic bipolar plate materials were successfully manufactured using laser cladding NiCr-based alloys with different Ti additions on a low carbon steel substrate. The titanium content within the coating was added, from 1.5 to 12.5 wt% Ti, and the variation in titanium was studied in terms of the microstructure and corrosion resistance. The study focused on electrochemically testing the coatings in a mild solution, such as 0.1 M Na_2_SO_4_ (acidulated with H_2_SO_4_ at pH = 5) with the addition of 0.1 ppm F^-^. The results led to the following conclusions:

- The microstructural investigation revealed a dendritic structure for all of the laser cladded coatings. Furthermore, as the Ti content increased within the coating, the size of the dendrites increased. The formation of precipitates and secondary phases was detected by EDX analysis and it was observed that the number of hard phases and the TiC particle size increased with the increase in Ti content within the coatings. The number of hard phases had a direct influence on the hardness measurement; thus, the coating containing 12.5% Ti presented the highest values.
- From the EIS measurements and potentiodynamic polarization, before measuring the current transients, it was noticed that a high content of Ti within the NiCr-base coating could provide a better corrosion resistance, up to the addition of 10% wt Ti. The corrosion current density of all coatings up to the addition of 10% wt Ti was less than DOE’s 2020 target of 1 µA·cm^−2^.
- It was noticed that the coating with the addition of 12.5% Ti addition presented a lower corrosion resistance due to the micro-cracks formed due to residual stress, which might be attributed to the higher amount of Ti present in the coating.
- Potentiostatic polarization measurements revealed that the coating with 10 wt% Ti addition had the lowest and most stable current density in the PEMFC anodic and cathodic environments, respectively.
- After measuring the current transients of the laser-cladded samples, the EIS measurements and potentiodynamic polarization were repeated. The results revealed that even if the corrosion resistance decreased after the durability test, the corrosion current density for all of the coatings in both environments was less than DOE’s 2020 target of 1 µA·cm^−2^. In conclusion, from the above results, we can say that the coating with the addition of 10 wt% Ti had great potential as a BP material for PEMFC applications.

## Figures and Tables

**Table 1:** Polarization parameters for laser-coated samples in the test solution, before the durability test.

Samples	i_corr_ [μA·cm^−2^]	E_corr_ [mV/Ag/AgCl]	−*b*_c_ [mV·dec^−1^]	*b*_a_ [mV·dec^−1^]	*R*_p_ [kΩ·cm^2^]
Substrate	2.814	−689	243	58	7
B	0.353	−158	292	176	135
B + 1.5% Ti	0.235	−165	257	144	171
B + 3% Ti	0.116	−179	212	178	362
B + 5% Ti	0.075	−199	198	168	526
B + 7% Ti	0.050	−221	184	152	723
B + 10% Ti	0.042	−241	203	121	784
B + 12.5% Ti	1.191	−342	176	150	30

**Table 2:** Calculated values of the circuit elements for modeling laser-cladded samples in the test solution, after potentiostatic polarization.

Samples	R_s_ [Ω·cm^−2^]	Y_0_ [S·cm^−2^·s^n^]	*n*	R_ct_ [Ω·cm^−2^]	Chi
B	47.56	2.93 × 10^−5^	0.87	4.64 × 10^4^	5.83 × 10^−3^
B + 1.5% Ti	46.87	3.02 × 10^−5^	0.88	6.92 × 10^4^	4.77 × 10^−3^
B + 3% Ti	49.50	3.33 × 10^−5^	0.90	9.98 × 10^4^	4.95 × 10^−3^
B + 5% Ti	47.12	3.64 × 10^−5^	0.91	2.65 × 10^5^	6.40 × 10^−3^
B + 7% Ti	47.77	3.87 × 10^−5^	0.92	7.07 × 10^5^	4.84 × 10^−3^
B + 10% Ti	47.08	4.91 × 10^−5^	0.92	7.95 × 10^5^	7.12 × 10^−3^
B + 12.5% Ti	47.41	1.66 × 10^−4^	0.84	3.85 × 10^4^	4.14 × 10^−3^

**Table 3:** Polarization parameters for laser-coated samples in the test solution after the durability test.

Samples	i_corr_ [μA·cm^−2^]	E_corr_ [mV/Ag/AgCl]	-*b*_c_ [mV·dec^−1^]	*b*_a_ [mV·dec^−1^]	*R*_p_ [kΩ·cm^2^]
**Cathodic environment**					
B	0.477	−315	182	134	70
B + 1.5% Ti	0.363	−308	176	122	86
B + 3% Ti	0.232	−298	183	130	142
B + 5% Ti	0.193	−276	188	119	164
B + 7% Ti	0.161	−245	186	123	200
B + 10% Ti	0.149	−236	172	120	206
**Anodic environment**					
B	0.868	−320	178	122	36
B + 1.5% Ti	0.623	−312	191	120	51
B + 3% Ti	0.468	−305	179	118	65
B + 5% Ti	0.213	−268	162	115	137
B + 7% Ti	0.185	−236	174	123	169
B + 10% Ti	0.177	−227	176	117	173

**Table 4:** Calculated values of the circuit elements for modeling the laser-coated samples in the test solution after potentiostatic polarization.

Samples	R_s_ [Ω·cm^−2^]	Y_0_ [S·cm^−2^·s^n^]	*n*	R_ct_ [Ω·cm^−2^]	Chi
**Cathodic environment**					
B	55.61	6.17 × 10^−4^	0.85	2.18 × 10^4^	1.02 × 10^−3^
B + 1.5% Ti	53.42	6.55 × 10^−4^	0.85	2.71 × 10^4^	2.73 × 10^−3^
B + 3% Ti	48.13	7.39 × 10^−4^	0.87	3.29 × 10^4^	1.91 × 10^−3^
B + 5% Ti	47.92	7.75 × 10^−4^	0.88	1.35 × 10^5^	2.09 × 10^−3^
B + 7% Ti	47.65	8.36 × 10^−4^	0.89	1.87 × 10^5^	1.78 × 10^−3^
B + 10% Ti	47.35	9.47 × 10^−4^	0.90	2.55 × 10^5^	1.53 × 10^−3^
**Anodic environment**					
B	46.93	2.68 × 10^−4^	0.77	1.16 × 10^4^	2.42 × 10^−3^
B + 1.5% Ti	49.87	2.87 × 10^−4^	0.82	1.41 × 10^4^	2.46 × 10^−3^
B + 3% Ti	48.51	2.96 × 10^−4^	0.87	1.86 × 10^4^	4.32 × 10^−3^
B + 5% Ti	47.43	3.11 × 10^−4^	0.89	1.98 × 10^4^	3.05 × 10^−3^
B + 7% Ti	47.58	3.14 × 10^−4^	0.90	2.04 × 10^4^	2.91 × 10^−3^
B + 10% Ti	46.62	3.53 × 10^−4^	0.91	2.33 × 10^4^	3.73 × 10^−3^

## Data Availability

Not applicable.
